# E-Health, another mechanism to recruit and retain healthcare professionals in remote areas: lessons learned from EQUI-ResHuS project in Mali

**DOI:** 10.1186/s12911-014-0120-8

**Published:** 2014-12-24

**Authors:** Cheick-Oumar Bagayoko, Marie-Pierre Gagnon, Diakaridia Traoré, Abdrahamane Anne, Abdel Kader Traoré, Antoine Geissbuhler

**Affiliations:** Centre d’Expertise et de Recherche en Télémédecine et E-Santé, CERTES, Bamako, Mali; Faculté de Médecine et d’Odonto-Stomatologie, FMOS, Bamako, Mali; Faculté des Sciences Infirmières, Université Laval, Quebec, Canada; Département de Radiologie et d’Informatique Médicale, Université de Genève, Geneva, Switzerland

## Abstract

**Background:**

The aim of this study was to evaluate the perceived influence of telehealth on recruitment and retention of healthcare professionals in remote areas in Mali.

**Methods:**

After 15 months of diagnosis imaging training and telehealth activities at four project sites in remote Mali, between May 2011 and August 2012, a 75-item questionnaire was administered to healthcare professionals to assess the various factors related to Information and Communication Technologies (ICT), especially telehealth, and their influence on health personnel recruitment and retention. Questions assessing perceived impact of telehealth on recruitment and retention of healthcare professionals were rated on a five-point Likert scale. Dependent variables were perceived influence of ICT on recruitment and retention and independent variables were access to ICT, ICT training, ICT use, perceived benefits and drawbacks of telehealth, and perceived barriers to recruitment and retention. A multiple linear regression was performed to identify variables explaining the respondents’ perceptions regarding telehealth influence on recruitment and retention.

**Results:**

Data analysis showed that professionals in remote areas have very positive perceptions of telehealth in general. Many benefits of telehealth for recruitment and retention were highlighted, with perceived benefits of ICT (p = 0.0478), perceived effects of telehealth on recruitment (p = 0.0018), telehealth training (0.0338) and information on telehealth (0.0073) being the strongest motivators for recruitment, while the perceived effects of telehealth on retention (p = 0.0018) was the only factor significantly associated with retention.

**Conclusions:**

Based on our study results, telehealth could represent a mechanism for recruiting and retaining health professionals in remote areas and could reduce the isolation of these professionals through networking opportunities.

**Electronic supplementary material:**

The online version of this article (doi:10.1186/s12911-014-0120-8) contains supplementary material, which is available to authorized users.

## Background

Equity in access to basic healthcare and access to qualified, and particularly to motivated, healthcare professionals remains a major challenge for many countries in sub-Saharan Africa [1].

This inequity of access to healthcare is even more accentuated in the case of medical specialties, which are almost nonexistent outside capital cities. For example in Mali there is only one radiologist outside Bamako, the capital, and no cardiologist within the country. Elsewhere a study shows that healthcare professionals who agree to go and serve in rural areas spend 50% of their time on the road to regional capitals for training related matters [2]. Thus the issue of motivated human resources and their retention is crucial to the balance and performance of the heath system, especially in countries such as Mali [3].

The problem of recruitment and retention of health professionals, especially in remote areas, has serious consequences for middle and low-income countries [4,5]. The shortage of qualified healthcare providers and their uneven distribution limits access to care for populations and contributes to increased mortality [6]. However, this situation is not specific to these countries only. For instance, the studies of Wade et al. in North Carolina, USA [7] and Johnston et al. in Ontario, Canada [8] exemplify the unequal distribution of the healthcare workforce in high-income countries.

As the study of Lehmann et al. [4] concludes, there is not a set answer to permanently solve the problem of attraction and retention of health professionals. To address this situation, several pilot projects in the field of ICT and health have been implemented. In Mali and sub-Saharan Africa several projects have demonstrated the potential role of innovative tools in providing equity of access to health care [9-11].

In line with these initiatives, the EQUI-ResHuS research project was initiated three years ago in Mali, with the aim to demonstrate how ICT applied to the health sector (i.e., eHealth) could contribute to making the health system more equitable [12]. The main activities in this project involved task shifting of medical imaging in obstetrics and cardiology to remote locations and the provision of Continuing Medical Education (CME) through distance learning.

The choice of task shifting of medical imaging was based on national priorities [13], given that medical specialties are scarce outside of the Malian capital, Bamako. While Mali is twice as large as France or Switzerland, patients in most of the nation are forced to travel hundreds or thousands of kilometres to undergo a simple ultrasound or electrocardiogram, hence the task-shifting choice in these fields.

The three-week training of healthcare professionals in ultrasound and cardiology consisted of basic practices. At the end of that period participating physicians and midwives should be able to conduct ultrasound and EKG procedures, distinguish abnormal from normal examinations and seek the advice of experts remotely through the tele-consultation platform.

For CME, online learning sessions were provided according to the needs expressed by health professionals from different sites of the project. The Dudal platform was used to accomplish that goal. The platform is a webcasting application developed through the activities of the RAFT telemedicine network (in French, *Réseau en Afrique Francophone pour la Télémédecine*) [14] and suited to low speed Internet.

The EQUI-ResHuS project is conducted in four district health centres: Dioila, Bankass, Djenné and Kolokani. The Dioila and Kolokani sites are located in the second region of Mali and are closest to the capital with relatively better roads. The sites of Bankass and Djénné are located in the fifth region in the north of the country and are thus more difficult to reach by road. Nevertheless, all these four sites have the same needs regarding deficits in infrastructure and qualified health personal. However, these sites are quite different in terms of size and health coverage (Table 1).

**Table 1:** **Information on the projects sites**

**Site**	**Superficy (km** ^**2**^ **)**	**Population**
Bankass	9504	263443
Dioila	7256	491210
Djenné	4651	207260
Kolokani	14380	233919

While there is limited research data about the role of telehealth in increasing access to specialized health care, several expert reports in developed countries, including the report of the French Ministry of Health, highlight the role that telehealth could play in healthcare organization [15]. In a study of the context of ICT and health conducted in Mali in 2010 under the EQUI-ResHuS project, 85% of respondents felt that the introduction of ICT had made their operations more efficient and 69% noted an improvement in their relationships with patients since they started using telehealth. In their view, this improvement was due to the speed and efficiency of diagnoses and the quality of medical information they collect from experts, all of which apparently reinforced trust and further reassured patients. These felt and lived benefits certainly explain the fact that 96% of respondents favored the introduction of ICT at all levels of the health pyramid in Mali [16].

The aim of our study was to evaluate the perceived influence of telehealth on recruitment and retention of healthcare professionals in remote areas in the context of the EQUI-ResHuS project.

## Methods

### Survey instrument

After a 15-month implementation phase, that took place between May 2011 and August 2012, a questionnaire modelled after another used in an earlier study in Quebec, Canada [17] and inspired by the Diffusion of Innovation Theory [18] was distributed to healthcare professionals in four district health centres that are part of EQUI-ResHuS project. This questionnaire is attached in Additional file 1.

The questionnaire contained a total of 75 items. Twenty-five items evaluated the healthcare workers’ knowledge and experience regarding ICT, while 37 items measured the respondents’ perceptions regarding the potential effect of telehealth on recruitment and retention of health professionals in remote areas, and 13 items measured respondents’ sociodemographic and professional characteristics. All items were measured on a five-point Likert scale, except sociodemographic data that were open or multiple-choice questions. Based on a previous validation of the instrument [17], the questionnaire was estimated to take about 30 minutes to complete. The study received approval from the “Réseau Informatique Malien d’Information et de Communication Médicale, REIMICOM” ethics committee.

Participants in the study were all health professionals and they signed the informed consent form included in the questionnaire.

### Description of constructs

The dependent variables were perceived influence of telehealth on recruitment and perceived influence of telehealth on retention, both measured by one item. The independent variables were access to ICT (3 items), information on ICT (3 items), ICT training (6 items), use of ICT last year (4 items), use of ICT last month (3 items), ICT usage by colleagues (3 items), perceived benefits of ICT (5 items), perceived effects on recruitment (12 items), perceived effects on retention (12 items), perceived barriers to recruitment (4 items) and perceived barriers to retention (4 items) (see Table 2).

**Table 2:** **Constitution and internal consistency of the constructs**

**Construct**	**Number of items**	**Cronbach’s alpha**	**Mean of the construct (SD)**
Access to ICT	3	0.73	1.97 (1.07)
Information on ICT	6	0.89	1.45 ( 0.76)
ICT training	6	0.89	1.92 (0.99)
Use of ICT last year	4	0.85	1.75 (0.99)
Use of ICT last months	3	0.83	1.70 ( 1.02)
ICT usage by colleagues	3	0.87	2.69 (1.32)
Perceived benefits of ICT	5	0.76	4.52 (0.72)
Perceived effect on recruitment	12	0.97	4.16 ( 0.98)
Perceived effect on retention	12	0.95	4.26 (0.74)
Barriers to recruitment	4	0.71	2.84 (1.03)
Barriers to retention	4	0.79	2.76 (1.24 )

Table 2 reports the construct validity of the scales, showing Cronbach’s alphas all above 0.70 which is considered acceptable according to Nunnally [19].

### Data analysis

Data were analyzed using SPSS software. We first explored relationships between telehealth experience (access, information, training, usage), perceptions about the advantages and disadvantages associated with the use telehealth and perceived impact of telehealth on recruitment and retention of professionals in remote areas through correlation analyses. We then assessed the influence of socio-demographic and professional characteristics on the dependent variables using non-parametric tests. Finally, we performed multiple linear regression analysis to determine which variables helped explain the participants’ perceptions concerning the influence of telehealth on 1) recruitment and 2) retention of health professionals in remote areas.

## Results

On a total of 45 questionnaires distributed to doctors, nurses, midwives, medical assistants and other staff involved in the delivery of healthcare in the four district health centres participating in the EQUI-ResHuS, 39 were completed, for a response rate of 86.7%.

### Socio-demographic characteristics

Males represented 84.6% of participants. A third of the participants were general practitioners, 28.2% were nurses, 12.9% were medical assistants, 7.7% were midwives, and 17.9% were classified as other professionals. The mean age of participants was 33.8 years, with a minimum of 25 years and a maximum of 58 years. The Table 3 below summarizes these socio-demographic characteristics.

**Table 3:** **Participants’ characteristics**

**Variable**		**Frequency**	**Percentage**
Sex	Female	6	15.4
	Male	33	84,6
Title	Generalist Doctor	13	33.3
	Nurse	11	28.2
	Medical Assistant	5	12.8
	Mid-wife	3	7.7
	Other	7	17.9
Birth place	Bamako	9	23
	Outside Bamako	30	77
Number of children (n = 34)	None	11	32.3
	1	7	20.6
	2	7	20.6
	3 or more	9	26.5
Age	Average		33.79 years
	min-max		25 - 58 years
Number of Years in the region	Average		5.13 years
	min-max		1–16 years

### ICT use and perceptions of the effects of telehealth

On average, respondents indicated that in a one-month period they spend 6.3% of the practice time in telehealth related activities, 12.4% of their time was allocated to the use of the Internet for clinical purposes, and 13.2% to the use of the Internet for educational purposes.

Access to continuing education and the possibility of receiving training delivered by multiple centres were the main perceived benefits for both recruitment and retention. The fact that telehealth contributed to promoting the health centre was also an important benefit for the recruitment and retention.

Perceived disadvantages were mainly the fear that telehealth would replace traveling for training purposes and was important for both recruitment and retention. Participants also considered as a disadvantage for recruitment the fact that telehealth can compete with the purchase of other materials for resource allocation The Table 4 summarizes the participant’s perceptions of ICT use and effects telehealth. The Table 5 shows the values of the dependent variables of interest: the influence of telehealth on recruitment and the influence of telehealth on retention.

**Table 4:** **Perception of the effects of telehealth (n = 39)**

**Perceived Benefits**	**Recruitment mean (SD)**	**Retention mean (SD)**
Accessibility to continuing medical education	4.41 (1.07)	4.44 (0.73)
Ability to share experiences in the field with other people	4.39 (1.05)	4.15 (1.00)
Availability of multi-centric training	4.25 (1,02)	4.26 (0.86)
Opportunity to promote the health center	4.24 (1.13)	4.32 (0.91)
Participation in team meetings remotely	4.22 (1.13)	4.17 (1.00)
Improved quality of practice	4.18 (1.20)	4.32 (0.97)
Quick access to specialized resources	4.16 (1.12)	4.25 (1.00)
Opportunity to provide and receive distance learning	4.16 (1.14)	4.11 (1.05)
Direct access to specialized resources	4.08 (1.17)	4.27 (0.90)
Continuity of services	3 .97 (1.28)	4.14 (0.88)
Possibility to have a second opinion	3.76 (1.21)	3.91 (1.15)
Better perception of patients	3.75 (1.20)	4.06 (0.94)
Perceived drawbacks	Recruitment	Retention
Telehealth replaces most trainings providing by external partners	3.28 (1.41)	3.18 (1.57)
Telehealth competes for resource allocation with the purchase of medical equipment	3.17 (1.47)	2.73 (1.51)
Telehealth replaces a doctor on site	2.62 (1.42)	2.79 (1.62)
Telehealth means a lack of staff	2.09 (1.29)	2.06 (1.43)

**Table 5:** **Values of the dependent variables**

**Variable**	**Mean**	**SD**
Influence of telehealth on recruitment	4.18	1.01
Influence of telehealth on the retention	3.70	1.27

### Regression analysis

Separate stepwise multiple linear regression analyses were performed to identify which constructs best explained health care professionals’ perception as regards the influence of telehealth on 1) recruitment and 2) retention in remote areas.

As shown in Table 6, four variables explained the perceived influence of telehealth on recruitment at the 5% level of significance: perceived telehealth effects on recruitment, perceived benefits of ICT, training and information. These variables explained 52% of the variance in perception of the influence of telehealth on recruiting professionals in remote areas (Table 5).

**Table 6:** **Final regression model for the variable impact of telehealth on recruitment**

**Variable**	**Estimated value of parameters**	**Standard error**	**t-test t**	**Pr > | t |**	**Estimated standard value**
Intercept	0.3495	0.9222	0.38	0.7073	0
Training	0.5228	0.2354	2.22	0.0338	0.473
Information	−0.5282	01838	−2.87	0.0073	−0.5577
Perceived benefits of ICT	0.4360	0.2116	2.06	0.0478	0.2843
Perceived telehealth effects on recruitment	0.5228	0.1531	3.41	0.0018	0.4720

R = 0.52, F = 8.25, p <0.0001.

As shown in Table 7, perceived benefits of telehealth on retention was the only factor that was significantly associated with perceived influence of telehealth on retention, at the 5% level. This variable explained 34% of the variance in perception of the impact of telehealth on the retention of professionals in remote areas.

**Table 7:** **Final regression model for the variable impact of telehealth on retention**

**Variable**	**Estimated parameters**	**Standard error**	**t-test**	**Pr > | t |**	**Estimated standard value**
Intercept	0.3495	0.9222	0.38	0.7073	0
Perceived telehealth effect on retention	0.5228	0.1531	3.41	0.0018	0.4720

R2 = 0.34, F = 17.64, p <0.001.

## Discussion

This study was conducted on four pilot sites participating in the EQUI-ResHuS project. Its aim was to evaluate the perceived influence of telehealth on recruitment and retention of healthcare professionals in remote areas in Mali. Participants were physicians and other healthcare professionals.

Our study shows that healthcare professionals generally have very positive perceptions of telehealth. They think that it could act as a lever for their recruitment and retention. Our results also show that the perceived influence of telehealth on recruitment is stronger than on retention. This could indicate that the perceived benefits of telehealth would have less weight in healthcare professionals’ decision to stay in a remote area than other factors such as family or salary for instance.

Similar to the studies by Mars [20,21], our results support the contention that perceived benefits of telehealth for the recruitment and retention of health professionals in remote areas are numerous, with the most important factors being access to training and the opportunity to interact with colleagues working at other health centres. Specifically, it would seem that telehealth is seen as a way to reduce the isolation of these professionals and to promote their networking.

Moreover, our results indicate that much needs to be done to increase access to ICT, to provide information about the potential role of ICT, and to increase the availability of ICT training. Unfortunately, only a small proportion of respondents was readily trained and provided with telehealth information, and few of them used ICT applications in their practice. Ultrasound and electrocardiogram training during this project was provided mainly for physicians working in participating centres. They could in turn train other healthcare professionals in their centre; however this was not always done. All professionals working in participating centres could nonetheless access distance education materials developed by the project. Our results also suggest that exposure and experience with telehealth also influenced the perceived benefit on recruitment and retention. Specifically, respondents who had been trained in the use of telehealth had a positive attitude towards telehealth and perceived its benefits for recruitment. They also had a more favourable perception of the importance of telehealth for recruitment. However, having simply received information on telehealth had a rather negative effect on healthcare professionals’ perception of the impact of telehealth on recruitment. This could be explained by the fact that more professionals were informed of the availability of telehealth in the centre, but were not trained to use it or did not have a chance to try it. In this sense, the results are consistent with the assumptions of Rogers’ Diffusion of Innovation model [18], which states that the adoption of an innovation is particularly influenced by the perception of its advantages, the triability of its results and the opportunity to try. This suggests that awareness of telehealth alone is insufficient to change perceptions, and that actual hands-on experience is likely required to influence behaviours and perceived benefits. As such, it may be appropriate to expand the training in the use of telehealth and ICT in general to all clinical staff of health centres involved in the EQUIResHus project to ensure uptake of the technology and maximize its impact.

Perceived impact of telehealth on the retention of health professionals in remote areas seems to be less important according to respondents. Telehealth perceived benefits was the only variable that could explain the perception of a positive impact of telehealth on retention. The percentage of explained variance was also lower than for recruitment; this suggests that other variables could affect the perception of the impact of telehealth on the retention of professionals in remote areas and that external factors may be more important in retaining healthcare providers in remote locations, including housing and social demographics.

This study has some limitations that could affect the interpretation of results. First, the use of a self-administered questionnaire may have induced social desirability bias, but we tried to limit this potential bias using an anonymous questionnaire. Second, there could be a selection bias if a greater proportion of professionals in favour of telehealth in fact participated in the study. However, the high response rate (87%) and the wide range in answers limited this potential bias. Third, the fact that some healthcare professionals received direct telehealth training while others did not may have influenced their perceptions, so it is difficult to draw strong conclusions from our results as both groups are considered together. Finally, we did not use a control group in this study so it is difficult to isolate the effect of the telehealth project that was taking place in the participating centres on the responses.

Further research is needed to acquire a better understanding of the potential impact of telehealth on healthcare professionals’ practice in remote areas, by exploring the factors that lead these professionals agree to work and remain in these areas. Concurrently, it is important to continue investigating the effects of telehealth at different levels of healthcare and along the training continuum to consolidate the evidence about the use of this technology in addressing human resource shortages affecting most LMIC. Telehealth may be particularly effective in attracting young professionals by leveraging the fact that telehealth allows them to keep their knowledge up to date and remain “connected” to their peers by networking and multi-centre training.

Finally, we learned during this research project that the training of healthcare professionals should also include ICT and telehealth in the curriculum so that future doctors, nurses, midwives and medical assistants are already familiar with these technologies when they graduate and join the workforce. For health centres that have implemented telehealth, the recruitment of professionals with prior ICT training would certainly be beneficial. In addition, the role of ICT should be included as part of CME, to reach a greater number of professionals.

## Conclusions

The EQUI-ResHuS project provided a unique context to study healthcare professionals’ perceptions regarding the influence of telehealth on their recruitment and retention in remote areas. Our results suggest that telehealth has a positive influence on recruitment and retention of isolated healthcare professionals.

Factors influencing perceived impact of telehealth on recruitment and retention identified in this study may inform the future scaling up of ICT technologies in Mali as well as other remote locations in Africa. Among these factors we can mention CME [22] and multi-centre collaborations and exchange of medical expertise with innovative tools.

However, a second phase of this study seems necessary to see how healthcare professionals’ perceptions can evolve over time and to assess the real impact of telehealth on recruitment and retention with a larger sample.

## Additional file

Additional file 1:
**Questionnaire.**
