# Supplementary material for: E-Health, another mechanism to recruit and retain healthcare professionals in remote areas: lessons learned from EQUI-ResHuS project in Mali
Source: BMC Med Inform Decis Mak. 2014 Dec 24;14:120. doi: 10.1186/s12911-014-0120-8 (PMC4305223; doi:10.1186/s12911-014-0120-8)
Supplement: Additional file 1: — Questionnaire. [file 12911_2014_120_MOESM1_ESM.doc]

**[
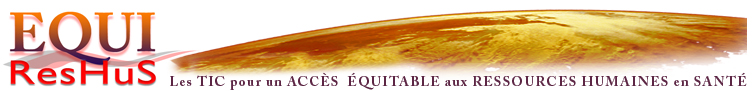
](http://www.certesmali.org/equireshus/)**

No du questionnaire

|  |  |  |
| --- | --- | --- |

#####

**La télésanté pour un accès Équitable aux Ressources Humaines en Santé qualifiées, motivées et bien soutenues en Afrique Francophone (EQUI-ResHuS)**

|  |
| --- |
| **DÉFINITION DES CONCEPTS UTILISÉS :** |
|  |
| ***Télésanté***: Désigne les soins et les services de santé, les services sociaux, préventifs ou curatifs, rendus à distance par le biais d’une télécommunication, incluant les échanges audiovisuels à des fins d’information, d’éducation et de recherche, et le traitement de données cliniques et administratives (Rapport de la Table ministérielle en télésanté du ministère de la Santé et des Services sociaux du Québec, 2001). |
| ***Téléformation*** : se définit comme « *l’enseignement professionnel à distance : il s’agit de la formation et de l’éducation continue dispensées aux différents professionnels de la santé* » (Rapport de la Table ministérielle en télésanté du ministère de la Santé et des Services sociaux du Québec, 2001). |
| ***Votre lieu de pratique :*** Il s’agit de **la structure de santé de santé où vous travaillez principalement en ce moment.** |
| **OBJECTIF DU PROJET :** |
|  |
| Ce projet vise à évaluer l’influence potentielle de la télésanté sur l’accès équitable aux professionnels de la santé et la pratique de ces derniers. |
|  |
| **BUT DU QUESTIONNAIRE :** |
|  |
| Recueillir votre opinion sur les facteurs influençant la pratique des professionnels de la santé dans les régions rurales, éloignées et isolées, ainsi que des facteurs associés à l’utilisation de la télésanté. Il s’agit savoir l’impact de la télésanté sur la motivation et la rétention de ces professionnels sur place. |
|  |
| **Marche à suivre :** |
|  |
| 1. Lire et **signer** le formulaire de consentement au verso. |
| 1. Pour chacune des questions contenues dans le questionnaire, cochez la réponse correspondant à ce que vous pensez. |
| 1. Se référer aux définitions. |
|  |
| Veuillez remettre le questionnaire et le formulaire de consentement dans l’enveloppe réponse qui vous est fournie et remettre le tout à la personne responsable de l’enquête. |

Ce projet de recherche est financé par : Le Centre de Recherche et de Développement International (CRDI),

Chercheurs responsables  Cheikh Oumar Bagayoko, MD, PhD Marie-Pierre Gagnon, PhD

de l’enquête Chercheur Principal EQUI-ResHuS Professeure

Faculté des Sciences Infirmières

Université Laval

### FORMULAIRE DE CONSENTEMENT

Projet de recherche

**« La télésanté pour un accès Équitable aux Ressources Humaines en Santé qualifiées, motivées et bien soutenues en Afrique Francophone (EQUI-ResHuS)**

**»**

**NATURE DE LA PARTICIPATION :**

- La participation au présent projet consiste à répondre à un questionnaire d’une durée approximative de dix minutes.
- Il est entendu que votre participation est tout à fait libre. Vous pourrez à n’importe quel moment mettre un terme à votre participation sans avoir à vous justifier ni à subir de préjudice quelconque.

**CONFIDENTIALITÉ ET ANONYMAT :**

- Les noms des participants ne paraîtront sur aucun rapport.
- Les questionnaires seront identifiés à l’aide d’un code de participation. Ce code de participation pourra être utilisé afin de faire correspondre vos réponses, advenant le cas où vous remplissiez de nouveau un questionnaire dans le cadre de ce projet de recherche.
- Seuls les membres de l’équipe de recherche auront accès à la liste des noms et des codes.
- Au terme de l’étude, les données brutes seront détruites. Seuls les fichiers informatisés, contenant des informations qui ne peuvent être identifiées aux participants, seront conservées aux fins d’analyses ultérieures.
- En aucun cas, les résultats individuels et les opinions des participants ne seront communiqués à qui que ce soit.

**QUESTIONS :**

- Pour toute question relative à ce projet de recherche, veuillez contacter :

Le Dr. Cheick Oumar BAGAYOKO, mail : [cob281@yahoo.fr](mailto:cob281@yahoo.fr)

Je soussigné(e) _______________________________ consens librement à participer au projet de recherche intitulé « La télésanté pour un Accès équitable aux Ressources Humaines en santé qualifiées, motivées et bien soutenues en Afrique Francophone (EQUI-ResHuS) ».

Je soussigné(e) _______________________________ consens librement à être contacté(e) de nouveau lors d’une étape ultérieure du projet de recherche intitulé « La télésanté : une stratégie pour soutenir les professionnels de la santé ».

_________________________________ ___________________

Signature du (de la) participant(e) Date

**No du questionnaire :**

|  |  |  |
| --- | --- | --- |

#####

1. Dans le cadre de votre pratique professionnelle, avez-vous accès à :

|  | **Jamais** | **Rarement** | **Parfois** | **Souvent** | **Toujours** |  |
| --- | --- | --- | --- | --- | --- | --- |
| 1. Internet | *1* | *2* | *3* | *4* | *5* | *Q1a* |
| 1. Télésanté | *1* | *2* | *3* | *4* | *5* | *Q1b* |
| 1. Téléformation | *1* | *2* | *3* | *4* | *5* | *Q1c* |

1. Avez-vous déjà reçu de la formation sur :

|  | **Jamais** | **Rarement** | **Parfois** | **Souvent** | **Très souvent** |  |
| --- | --- | --- | --- | --- | --- | --- |
| 1. L’utilisation d’Internet | *1* | *2* | *3* | *4* | *5* | *Q2a* |
| 1. L’utilisation de la télésanté | *1* | *2* | *3* | *4* | *5* | *Q2b* |
| 1. L’utilisation de la téléformation | *1* | *2* | *3* | *4* | *5* | *Q2c* |

1. Dans les 3 dernières années, avez-vous participé à un symposium, congrès, colloque ou séminaire portant sur :

|  | **Jamais** | **Rarement** | **Parfois** | **Souvent** | **Très souvent** |  |
| --- | --- | --- | --- | --- | --- | --- |
| 1. L’utilisation d’Internet | *1* | *2* | *3* | *4* | *5* | *Q3a* |
| 1. L’utilisation de la télésanté | *1* | *2* | *3* | *4* | *5* | *Q3b* |
| 1. L’utilisation de la téléformation | *1* | *2* | *3* | *4* | *5* | *Q3c* |

1. Dans les 3 dernières années, avez-vous participé à des réunions professionnelles où il a été question de :

|  | **Jamais** | **Rarement** | **Parfois** | **Souvent** | **Très souvent** |  |
| --- | --- | --- | --- | --- | --- | --- |
| 1. L’utilisation d’Internet | *1* | *2* | *3* | *4* | *5* | *Q4a* |
| 1. L’utilisation de la télésanté | *1* | *2* | *3* | *4* | *5* | *Q4b* |
| 1. L’utilisation de la téléformation | *1* | *2* | *3* | *4* | *5* | *Q4c* |

1. Vous arrive-t-il de consulter vos collègues sur :

|  | **Jamais** | **Rarement** | **Parfois** | **Souvent** | **Très souvent** |  |
| --- | --- | --- | --- | --- | --- | --- |
| 1. L’utilisation d’Internet | *1* | *2* | *3* | *4* | *5* | *Q5a* |
| 1. L’utilisation de la télésanté | *1* | *2* | *3* | *4* | *5* | *Q5b* |
| 1. L’utilisation de la téléformation | *1* | *2* | *3* | *4* | *5* | *Q5c* |

1. Dans la dernière année :

|  | **Jamais** | **Rarement** | **Parfois** | **Souvent** | **Très souvent** |  |
| --- | --- | --- | --- | --- | --- | --- |
| 1. Vous avez utilisé la télésanté | *1* | *2* | *3* | *4* | *5* | *Q6a* |
| 1. Vous avez reçu de la téléformation | *1* | *2* | *3* | *4* | *5* | *Q6b* |
| 1. Vous avez utilisé Internet à des fins cliniques | *1* | *2* | *3* | *4* | *5* | *Q6c* |
| 1. Vous avez suivi de la formation provenant du Réseau en Afrique Francophone pour la Télémédecine (RAFT) | *1* | *2* | *3* | *4* | *5* | *Q6d* |

1. Dans le dernier mois,,combien de fois avez-vous utilisé les sources suivantes dans votre pratique clinique :

|  | **Jamais** | **Rarement** | **Parfois** | **Souvent** | **Très souvent** |  |
| --- | --- | --- | --- | --- | --- | --- |
| 1. Internet | *1* | *2* | *3* | *4* | *5* | *Q7a* |
| 1. Télésanté | *1* | *2* | *3* | *4* | *5* | *Q7b* |
| 1. Information provenant d’une téléformation | *1* | *2* | *3* | *4* | *5* | *Q7c* |

1. Travaillez-vous avec des collègues qui utilisent :

|  | **Jamais** | **Rarement** | **Parfois** | **Souvent** | **Très souvent** |  |
| --- | --- | --- | --- | --- | --- | --- |
| 1. Internet | *1* | *2* | *3* | *4* | *5* | *Q8a* |
| 1. Télésanté | *1* | *2* | *3* | *4* | *5* | *Q8b* |
| 1. Information provenant d’une téléformation | *1* | *2* | *3* | *4* | *5* | *Q8c* |

1. Dans un mois normal de travail, quel est la proportion de votre temps de pratique où vous utilisez :
   1. La télésanté ?____________% de votre temps de pratique *Q9a*
   2. Internet à des fins cliniques ? ____________% de votre temps de pratique *Q9b*
   3. Internet à des fins éducatives ? ____________% de votre temps de pratique *Q9c*
2. En tenant compte de vos principales activités de formations continues de la dernière année, quel % de vos formations se sont faites :
   1. par visioconférence ? ____________% de vos formations. *Q10a*
   2. par Internet ? ____________% de vos formations. *Q10b*
   3. par conférencier invité ? ____________% de vos formations. *Q10c*
   4. par des rencontres à l’extérieur ? ____________% de vos formations. *Q10d*
3. Selon vous, l’utilisation des technologies de l’information et des communications (TIC) (télésanté, téléformation, internet) dans votre pratique professionnelle est :

|  | **Jamais** | **Rarement** | **Parfois** | **Souvent** | **Toujours** | **Ne s’applique pas (NAP)** |  |
| --- | --- | --- | --- | --- | --- | --- | --- |
| 1. Pertinente | *1* | *2* | *3* | *4* | *5* | *6* | *Q11a* |
| 1. Un moyen efficace d’obtenir de l’information | *1* | *2* | *3* | *4* | *5* | *6* | *Q11b* |
| 1. Un moyen d'accéder aux connaissances dont vous avez besoin dans votre travail | *1* | *2* | *3* | *4* | *5* | *6* | *Q11c* |
| 1. Adaptée à votre travail | *1* | *2* | *3* | *4* | *5* | *6* | *Q11d* |
| 1. Soutenue par des moyens techniques adéquats | *1* | *2* | *3* | *4* | *5* | *6* | *Q11e* |

1. Les relations de travail que vous établissez au moyen des TIC (télésanté, téléformation, Internet) se font-elles :

|  | **Jamais** | **Rarement** | **Parfois** | **Souvent** | **Toujours** | **NAP** |  |
| --- | --- | --- | --- | --- | --- | --- | --- |
| 1. avec les hôîtaux nationaux | *1* | *2* | *3* | *4* | *5* | *6* | *Q12a* |
| 1. avec les hôpitaux de district ou des centres de référence | *1* | *2* | *3* | *4* | *5* | *6* | *Q12b* |

1. Dans quelle mesure chacun des éléments suivants, associés à l’utilisation de la télésanté, pourrait motiver votre choix de lieu de pratique :

| **FACTEURS** | **Pas du tout**  **important** | **Un peu important** | **Plus ou moins important** | **Assez important** | **Très important** |  |
| --- | --- | --- | --- | --- | --- | --- |
| 1. L’accessibilité directe aux ressources spécialisées | *1* | *2* | *3* | *4* | *5* | *Q13a* |
| 1. La possibilité d’avoir une seconde opinion | *1* | *2* | *3* | *4* | *5* | *Q13b* |
| 1. La rapidité d’accès aux ressources spécialisées | *1* | *2* | *3* | *4* | *5* | *Q13c* |
| 1. L’amélioration de la qualité de la pratique | *1* | *2* | *3* | *4* | *5* | *Q13d* |
| 1. La meilleure impression des patients | *1* | *2* | *3* | *4* | *5* | *Q13e* |
| 1. La continuité des services | *1* | *2* | *3* | *4* | *5* | *Q13f* |
| 1. L’accessibilité à la formation médicale continue | *1* | *2* | *3* | *4* | *5* | *Q13g* |
| 1. La participation à des réunions d’équipe à distance | *1* | *2* | *3* | *4* | *5* | *Q13h* |
| 1. L’accessibilité à des formations multi-centres | *1* | *2* | *3* | *4* | *5* | *Q13i* |
| 1. La possibilité de programme de formation bidirectionnelle à distance | *1* | *2* | *3* | *4* | *5* | *Q13j* |
| 1. La possibilité de promouvoir le centre de santé | *1* | *2* | *3* | *4* | *5* | *Q13k* |
| 1. La possibilité de partager ses expériences du terrain avec les autres | *1* | *2* | *3* | *4* | *5* | *Q13l* |

1. Existe-t-il d’autres éléments associés à l’utilisation de la télésanté qui auraient pu être liés à votre choix de lieu de pratique ? *Q14*

______________________________________________________________________________________________________________________________________________________________________________________________________________________________________________________________________________________________________________________________________________________

1. Dans quelle mesure chacun des éléments suivants associés à l’utilisation de la télésanté pourrait être lié à votre choix de **maintenir** votre lieu de pratique actuel :

| **FACTEURS** | **Pas du tout**  **important** | **Un peu important** | **Plus ou moins important** | **Assez important** | **Très important** |  |
| --- | --- | --- | --- | --- | --- | --- |
| 1. L’accessibilité directe aux ressources spécialisées | *1* | *2* | *3* | *4* | *5* | *Q15a* |
| 1. La possibilité d’avoir une seconde opinion | *1* | *2* | *3* | *4* | *5* | *Q15b* |
| 1. La rapidité d’accès aux ressources spécialisées | *1* | *2* | *3* | *4* | *5* | *Q15c* |
| 1. L’amélioration de la qualité de la pratique | *1* | *2* | *3* | *4* | *5* | *Q15d* |
| 1. La meilleure impression des patients | *1* | *2* | *3* | *4* | *5* | *Q15e* |
| 1. Lacontinuité des services | *1* | *2* | *3* | *4* | *5* | *Q15f* |
| 1. L’accessibilité à la formation médicale continue | *1* | *2* | *3* | *4* | *5* | *Q15g* |
| 1. La participation à des réunions d’équipe à distance | *1* | *2* | *3* | *4* | *5* | *Q15h* |
| 1. L’accessibilité à des formations multi-centres | *1* | *2* | *3* | *4* | *5* | *Q15i* |
| 1. L’organisation de programme de formation bidirectionnelle (le site éloigné donne la formation) | *1* | *2* | *3* | *4* | *5* | *Q15j* |
| 1. La possibilité de promouvoir le centre de santé | *1* | *2* | *3* | *4* | *5* | *Q15k* |
| 1. La possibilité de faire de l’enseignement | *1* | *2* | *3* | *4* | *5* | *Q15l* |

1. Existe-t-il d’autres éléments associés à l’utilisation de la télésanté qui pourrait être liés à votre choix de **maintenir** votre lieu de pratique ?

_______________________________________________________________________________________________________________*Q16*____________________________________________________________________________________________________________________________________________________________________________________________________________________________________

1. À quel point chacune des conditions suivantes pourraient **nuire** au recrutement de professionnels de la santé en région éloignée :

| **FACTEURS** | **Pas du tout**  **important** | **Un peu important** | **Plus ou moins important** | **Assez important** | **Très important** |  |
| --- | --- | --- | --- | --- | --- | --- |
| 1. La télésanté remplace un médecin sur place | *1* | *2* | *3* | *4* | *5* | *Q17a* |
| 1. La télésanté remplace la majorité des formations à l’extérieur | *1* | *2* | *3* | *4* | *5* | *Q17b* |
| 1. La télésanté signifie un manque de personnel | *1* | *2* | *3* | *4* | *5* | *Q17c* |
| 1. La télésanté entre en compétition dans l’allocation des ressources pour l’achat d’équipement médical | *1* | *2* | *3* | *4* | *5* | *Q17d* |

1. Existe-t-il d’autres conditions associées à l’utilisation de la télésanté qui pourraient **nuire** au recrutement de professionnels de la santé en région éloignée ?

_______________________________________________________________________________________________________________*Q18*____________________________________________________________________________________________________________________________________________________________________________________________________________________________________

1. À quel point chacune des conditions suivantes pourrait **nuire** à la rétention des professionnels de la santé en région éloignée :

| **FACTEURS** | **Pas du tout**  **important** | **Un peu important** | **Plus ou moins important** | **Assez important** | **Très important** |  |
| --- | --- | --- | --- | --- | --- | --- |
| 1. La télésanté remplace un médecin sur place | *1* | *2* | *3* | *4* | *5* | *Q19a* |
| 1. La télésanté remplace la majorité les formations à l’extérieur | *1* | *2* | *3* | *4* | *5* | *Q19b* |
| 1. La télésanté signifie manque de personnel | *1* | *2* | *3* | *4* | *5* | *Q19c* |
| 1. La télésanté entre en compétition dans l’allocation des ressources pour l’achat d’équipement médical | *1* | *2* | *3* | *4* | *5* | *Q19d* |

1. Existe-t-il d’autres conditions associées à l’utilisation de la télésanté qui pourraient **nuire** à la rétention des professionnels de la santé en région éloignée ? *Q20*

______________________________________________________________________________________________________________________________________________________________________________________________________________________________________________________________________________________________________________________________________________________

1. Selon vous, est-ce que la télésanté aurait pu être un facteur de motivation important dans votre choix de lieu de pratique ? *Q21*

| **Très important** *5* | **Assez important** *4* | **Plus ou moins important** *3* | **Peu important** *2* | **Pas du tout important** *1* |
| --- | --- | --- | --- | --- |

1. Selon vous, est-ce que la télésanté pourrait être un facteur important d’influence sur votre choix de maintenir votre lieu de pratique ? *Q22*

| **Très important** *5* | **Assez important** *4* | **Plus ou moins important** *3* | **Peu important** *2* | **Pas du tout important** *1* |
| --- | --- | --- | --- | --- |

1. Quel type d’application serait le plus utile dans votre pratique?

| **APPLICATIONS** | **Inutile** | **Peu utile** | **Plus ou moins utile** | **Assez utile** | **Très utile** | **NAP** |  |
| --- | --- | --- | --- | --- | --- | --- | --- |
| 1. Télé-echographie | *1* | *2* | *3* | *4* | *5* | *6* | *Q23a* |
| 1. Télécardiologie | *1* | *2* | *3* | *4* | *5* | *6* | *Q23b* |
| 1. Formation médicale continue à distance | *1* | *2* | *3* | *4* | *5* | *6* | *Q23c* |
| 1. Système d’aide à la décision médicale et formation | *1* | *2* | *3* | *4* | *5* | *6* | *Q23d* |
| 1. Téléconsultation / Télé-expertise | *1* | *2* | *3* | *4* | *5* | *6* | *Q23e* |

Autres applications : ________________________________________________________________________________________________ *Q23i*

1. Selon vous, quelles seraient les 2 conditions les plus importantes qui favoriseraient l'utilisation de la télésanté dans votre pratique? *Q24*

1)____________________________________________________________________________________________________________

2)____________________________________________________________________________________________________________

1. Selon vous, quelles seraient les 2 conditions les plus importantes qui favoriseraient l’intégration de la télésanté au fonctionnement habituel de votre centre de santé ? *Q25*

1)____________________________________________________________________________________________________________

2)____________________________________________________________________________________________________________

1. Quel est votre âge ? **____________________** *Q26*
2. Quel est votre genre ? **Féminin** *1* ; **Masculin** *2 Q27*
3. Votre lieu de naissance ?_______________________ *Q28a* Le lieu de naissance de votre conjoint-e ?__________________NAP  *Q28b*
4. Nombre d’enfant-s :______ *Q29*
5. Niveau scolaire des enfants ? préscolaire (0-5 ans) *1* ; primaire (6-12 ans) *2* ; secondaire (13-17ans) *3*; post-secondaire *4*; NAP *5 Q30*
6. Êtes-vous … ? **Médecin généraliste**  *1*; **Médecin spécialiste** *2* **Spécialité  :_________________________________** *Q31b*

*Q31a* **Sage femme** *3* **Infirmier/ère** *4* **Assistant médical** *5*

**Autre** *6*  **préciser :** ____________________________________________________________ *Q31c*

1. À quelle institution avez-vous obtenu votre diplôme de professionnel de la santé ? ______________________________________ *Q32*
2. À part votre diplôme de professionnel de la santé, détenez-vous un ou d’autres diplômes universitaires? **Oui** *1***; Non** *2 Q33a*

Si oui, s.v.p. veuillez indiquer lequel/lesquels :

**License**  *1* **; Doctorat** *2* **; Maîtrise** *3***; Autres** *4* *Q33b* ***préciser****______________________________ Q33c*

Dans ce centre

1. Depuis combien d’années pratiquez-vous dans cette région ? ________________ *Q35*
2. À l’exclusion du temps de garde en disponibilité (c’est-à-dire le temps en dehors de l’horaire habituel de vos activités où vous êtes disponible pour les patients), combien d’heures par semaine consacrez-vous en moyenne à vos activités professionnelles ? __________heures/semaine *Q36*
3. Combien d’heures par semaine consacrez-vous en moyenne à la garde?
4. Sur appel :_______________heures/semaines
5. Sur place :_______________heures/semaines *Q37*
6. Taille de votre équipe de travail immédiate (vous incluant) ? ____________médecins *Q38*

**Merci de votre précieuse collaboration !**

Tous vos commentaires tant sur le questionnaire que sur la télésanté dans votre pratique sont les bienvenus :

____________________________________________________________________________________________________________________________________________________________________________________________________________________________________________________________________________________________________________________________________________________________________________________________________________________________________________________________________________________________________________________________________________________________________________________________________________________________________________________________________________________________________________________________________________________________________________________________________________________________________________________________________________________________________________________________________________________________________________________________________________________________________________________________________________________________________________________________________________________________________________________________________________________________________________________________________________________________________________________________________________________________________________________________________________________________________________________________________________________________________________________________________________________________________________________________________________________________________________________________

__________________________________________________________________________________________________________________________________________________________________________________________________________________________________________________________________________________________________________________________________________________________________________________________________________________________________________________________________________________________________________________________________________________________________________________________________________________________________________________________________________________________________________________________________________________________________________________________________________________________________________________________________________________________________________________________________________________________________________________________________________________________________________________________________________________________________________________________________________________________________________________________________________________________________________________________________________________________________________________________________________________________________________________________________________________________________________________________________________________________________________________________________________________________________________________________________________________________________________________________________________________________________________________________________________________________________________________________________________________________________________________________________________________________________________________________________________________________________________________________________________________
